# CYP2C19 polymorphism and coronary in-stent restenosis: A systematic review and meta-analysis

**DOI:** 10.12688/f1000research.109321.3

**Published:** 2025-01-15

**Authors:** Yusra Pintaningrum, Vitriyaturrida, Ivana Purnama Dewi, Hendy Bhaskara Perdana Putra, Idar Mappangara, Muzakkir Amir, Irawan Yusuf, Agussalim Bukhari

**Affiliations:** 1 Department of Cardiology and Vascular Medicine, Faculty of Medicine, University of Mataram, Mataram, West Nusa Tenggara, 83126, Indonesia; 2 Department of Cardiology and Vascular Medicine, Slamet Martodirdjo General Hospital, Pamekasan, Madura, 69317, Indonesia; 3 Department of Cardiology and Vascular Medicine, Faculty of Medicine, Airlangga University - Dr Soetomo General Hospital, Surabaya, East Java, 60132, Indonesia; 4 Department of Cardiology and Vascular Medicine, Faculty of Medicine, Duta Wacana Christian University, Yogyakarta, Special Region of Yogyakarta, 55224, Indonesia; 5 Emergency Department, dr. Ramelan Navy Hospital, Surabaya, East Java, 60244, Indonesia; 6 Department of Cardiology and Vascular Medicine, Faculty of Medicine, University of Hasanuddin, Makasar, South Sulawesi, 90245, Indonesia; 7 Department of Physiology, Faculty of Medicine, University of Hasanuddin, Makassar, South Sulawesi, 90245, Indonesia; 8 Department of Nutrition, Faculty of Medicine, University of Hasanuddin, Makassar, South Sulawesi, 90245, Indonesia

**Keywords:** CYP2C19, polymorphism, coronary in-stent restenosis, systematic review, clopidogrel

## Abstract

**Background:**

In-stent restenosis (ISR) remains a major drawback in coronary stenting. The association between the CYP2C19 loss of function (LOF) gene and the prevalence of ISR after coronary stenting remains controversial. Previous studies have produced conflicting results and have been limited by their small population sizes. We conducted this systematic review and meta-analysis to determine the association between the presence of the CYP2C19 LOF gene and the prevalence of ISR.

**Methods:**

A systematic online database search was performed until April 2021. The primary outcome was ISR and assessed using OR with 95% CI. Quality of the study was assessed using the Newcastle Ottawa Scale. *I ^2^* was applied to examine heterogeneities among the studies.

**Results:**

A total of 284 patients (four non-randomized controlled trial studies) were included in this study. Two hundred and six patients had wild-type genotypes, while 78 patients had the LOF genotype. Among the 78 patients with the LOF gene, 38 patients had an ISR. Meanwhile, of the 206 patients with a wild-type gene, 69 patients had an ISR. LOF gene was associated with a higher risk of ISR (OR 95% CI = 2.71 [1.42–5.16], P = 0.003). However, study-specific variability should be considered when applying these findings clinically.

**Conclusions:**

Patients with LOF genes, regardless of the allele variation, treated with clopidogrel, had a higher likelihood of ISR after coronary stenting.

## Introduction

Despite the fact that the prevalence of in-stent restenosis (ISR) has decreased gradually, consistent with stent evolution, ISR remains a major drawback in coronary stenting. ^1^ ISR is defined as stenosis with a diameter > 50% at the stent segment or its edges. In the stent era, ISR is primarily a result of neointimal hyperplasia. Stent implantation causes injury to endothelial cells and induces several complex biological responses, including the activation, proliferation, and migration of smooth muscle cells (SMC) into the endovascular lumen. Vascular smooth muscle cells (VSMCs) play a crucial role in the pathogenesis of vascular diseases, including vascular inflammation and restenosis following angioplasty. ^2^ Stent implantation also stimulates the release of thrombogenic and vasoactive cytokines. ^3^

An animal study conducted by Niu *et al*. revealed that expression of the P2Y12 receptor in the vessel wall promoted atherogenesis and VSMC migration from media to intima. Direct activation of P2Y12 also mediates VSMC proliferation. ^4^ Furthermore, thrombin-induced P2Y12 is known to enhance mitogenesis in human SMC. ^5^ Clopidogrel has long been used as a P2Y12 inhibitor.

Clopidogrel is converted to an active metabolite by the enzyme CYP2C19. The genes encoding the CYP enzyme are polymorphic, and several variants have been related to increased or decreased activity of the drug. Based on the genetic polymorphism of CYP2C19, wild-type homozygote CYP2C19*1*1 is a powerful metabolizer. However, carriers of CYP2C19 loss of function (LOF) alleles (*2, *3, *4, *5) are poorer metabolizers. ^6^ Several studies have been conducted to assess the association between ISR events and CYP2C19 polymorphism. ^7^^–^^10^ However, the results are conflicting. Therefore, this study aims to conduct a meta-analysis regarding the association between CYP2C19 LOF genes and ISR events.

## Methods

This study was conducted following Cochrane’s methodology and PRISMA guidelines. ^11^ It is registered in the PROSPERO database, registration number: 293424.

### Search strategy

We performed a systematic search of several online databases (Scopus, PubMed, Cochrane Central Register of Controlled Trials [CENTRAL], ScienceDirect, and ResearchGate) for all studies on CYP2C19 polymorphism and in-stent restenosis published between January 2012 and December 2021. We used the terms “coronary restenosis” AND “in-stent restenosis” AND “gene polymorphism” AND “CYP2C19” AND “genotype”.

### Eligibility criteria

The criteria included were observational studies or randomized controlled trials (RCTs), which examined the association between gene polymorphism and coronary restenosis after stent implantation. Exclusion criteria included case reports, case series, studies in languages other than English, and non-human studies. Two reviewers (V. and HBPP) independently screened the search results according to the inclusion and exclusion criteria. Any discrepancies were settled through discussion with a third investigator (YP) until a consensus was reached.

### Quality and publication bias assessment

The studies were evaluated based on Cochrane methodological criteria. However, the quality of observational studies was evaluated using the Newcastle – Ottawa Scale. ^12^ Funnel plot was used to assess the potential for publication bias.

### Endpoint

The primary endpoint was in-stent restenosis after stent implantation regardless of the type of previous stent.

### Statistical analysis

Statistical analysis was carried out using RevMan 5.4 software (RRID:SCR_003581). All outcomes were analyzed using a random-effects model and presented as odds ratios (OR) with 95% confidence intervals (CI). A P-value equal to or less than 0.05 was considered statistically significant. *I* ^2^ assessed heterogeneity. *I* ^2^ values less than 25% were defined as low heterogeneity; *I* ^2^ values between 25% and 50% were defined as moderate heterogeneity, and *I* ^2^ values greater than 50% were defined as high heterogeneity.

## Results

**Figure 1.**
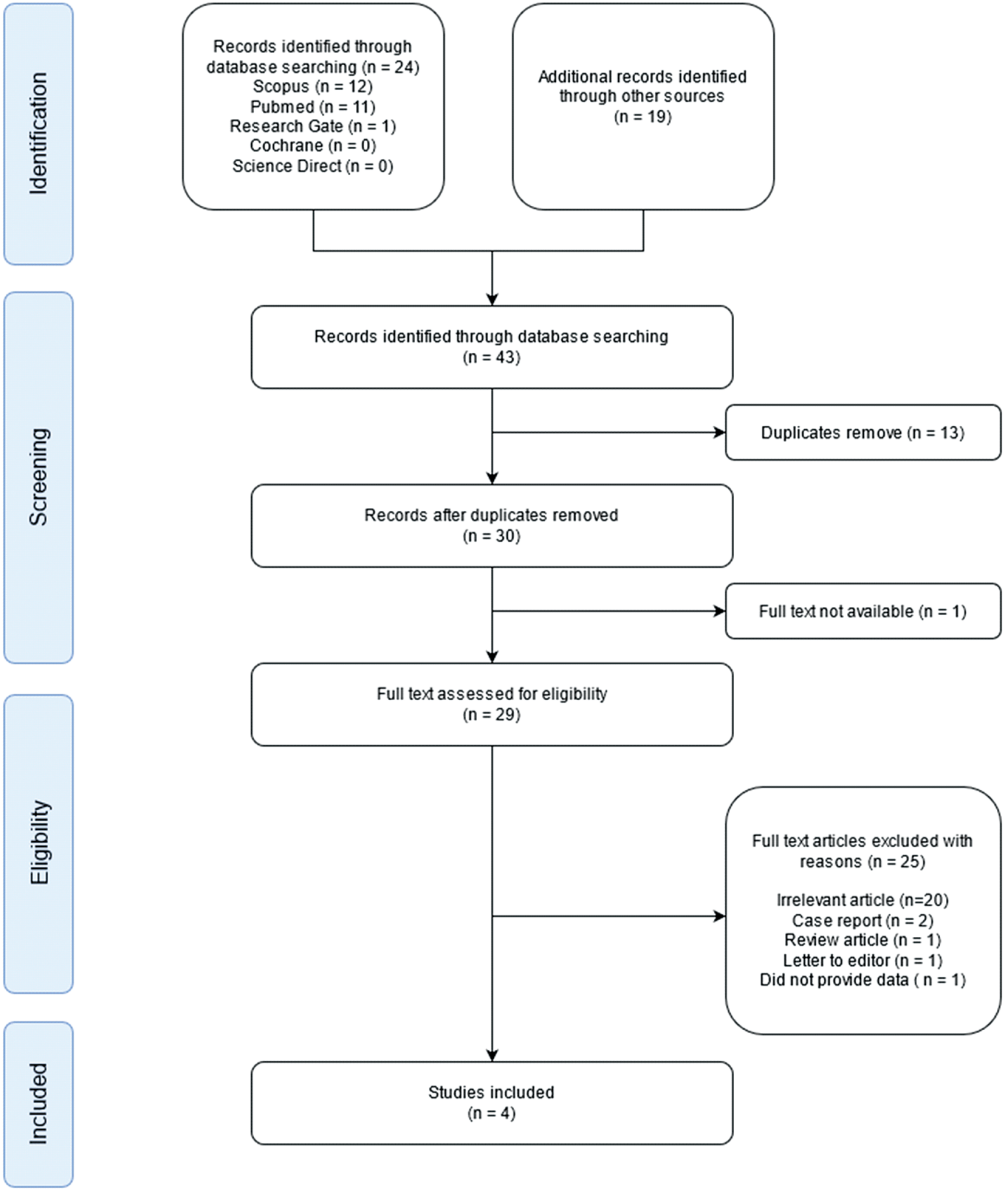
Preferred reporting items for systematic reviews and meta-analyses (PRISMA) flow diagram of study selection.

The initial search identified a total of 43 studies. Of these 43 studies, one article did not contain full text, and 13 studies were duplicates, leaving 29 studies. Of the remaining studies, 20 were excluded due to irrelevant titles or abstracts. Furthermore, five full-text articles were categorized as case reports, reviews, letters to the editor, or did not provide data for calculation. Finally, four observational studies were included in the qualitative synthesis ( Figure 1). A total of 284 patients were included in the study. Two hundred and six patients had wild-type genotypes, while 78 patients had a LOF genotype. The quality of the four observational studies is shown in Table 1. The funnel plot appears symmetrical distribution of studies suggesting that the results are likely robust and not significantly influenced by selective reporting.

**Table 1.** Newcastle – Ottawa scale indicating the quality of each included study.

	Selection	Comparability	Outcome	Total score	Overall grade
Da Costa *et al*. (2020) ^7^	*	0	**	3	Poor
Zhang *et al*. (2020) ^8^	**	*	**	5	Fair
Wirth *et al*. (2018) ^9^	**	*	***	6	Fair
Nozari *et al*. (2015) ^10^	**	*	***	6	Fair

Asterisks indicate the star rating according to the Newcastle-Ottawa Scale.
- Good quality: 3 or 4 stars in selection domain AND 1 or 2 stars in comparability domain AND 2 or 3 stars in outcome domain. - Fair quality: 2 stars in selection domain AND 1 or 2 stars in comparability domain AND 2 or 3 stars in outcome domain. - Poor quality: 0 or 1 star in selection domain OR 0 stars in comparability domain OR 0 or 1 stars in outcome domain

### Patient characteristics

The patients included in the studies had similar characteristics; they were predominantly male (62–72%) ( Table 2), with a mean age range of 60–66 years, except for the study by Da Costa *et al*., which did not report their patients’ characteristics. ^7^ The patients in the Wirth *et al*. study had the highest proportion of diabetes and hypertension compared to the other studies. ^9^

**Table 2.** Included studies and baseline characteristics.

**Author (year)**	Da Costa *et al*. (2020) ^7^	Zhang *et al*. (2020) ^8^	Wirth *et al*. (2018) ^9^	Nozari *et al*. (2015) ^10^
**Study type**	Cross-sectional	Retrospective cohort	Retrospective cohort	Case match study
**Sample size (n)**	24	78	82	100
**Wild gene (%)**	6 (25.0)	51 (65.4)	60 (73.2)	89 (89)
**LOF gene (%)**	18 (75.0)	27 (34.6)	22 (26.8)	11 (11)
**LOF gene type**	*1*2/ *2*2	alelle *2 or *3	alelle *2	*1*2
**Stent type**	Not clear	DES	BMS & DES	BMS & DES
**Clopidogrel dose**	Not clear	75 mg/day	Not clear	75 mg/day
**Total ISR event**	13	17	29	50
**BMS – ISR**	Not clear	N/A	4	30
**DES – ISR**	Not clear	17	25	20
**Age (mean ± SD, years)**	N/A	66.69 ± 6.2	64.58 ± 9.2	60.09 ± 10.29
**Male (%)**	N/A	49 (62.8)	65 (79.2)	72 (72)
**Diabetes mellitus (%)**	N/A	25 (32.0)	49 (59.8)	26 (26)
**Hypertension (%)**	N/A	54 (69.2)	74 (90.2)	51 (51)
**Dyslipidemia (%)**	N/A	N/A	76 (92.7)	62 (62)
**Smoking (%)**	24 (100)	32 (41.0)	20 (24.4)	20 (20)

Abbreviations: LOF: loss of function; ISR: In-stent restenosis; BMS: bare metal stent; DES: drug eluting stent.

### Genotype characteristics

In most of the studies examined, the wild gene (*1*1) was dominant (in 65%–89% of patients) ( Table 2). Only in the study by Da Costa *et al*. was the wild gene the minority (in only 25% of patients). ^7^ The four selected studies examined different LOF genes. The study by Nozari *et al*. showed the heterozygote *1*2 LOF gene. ^10^ In contrast, the study by Da Costa *et al*. examined LOF heterozygote *1*2 and homozygote *2*2 genes without clearly specifying the percentage. ^7^ The study by Zhang *et al*. only mentioned that the LOF gene has a *2 or *3 allele. ^8^ The study by Wirth *et al*. stated that the LOF gene has a *2 allele. ^9^

### Stent characteristics

Da Costa *et al*. did not state clearly the stent type used in their study. ^7^ However, Zhang *et al*. only used drug-eluting stents (DES). ^8^ Meanwhile, Wirth *et al*. and Nozari *et al*. used bare-metal stents (BMS) and DES in their studies without clearly stating the percentages ( Table 2). ^9^^,^^10^

### Reported outcomes

**Figure 2.**
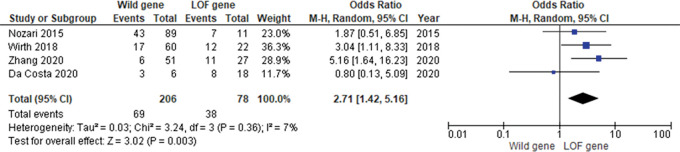
Pooled analysis shows a statistically significant association between the LOF gene and higher ISR incidence.

From all of the studies, the only incidence of ISR can be calculated quantitatively. However, regardless of the time of presentation from the stent implantation to ISR detection, it is known that there were 38 incidents of ISR in the 78 patients with LOF genes and 69 incidents of ISR in the 206 patients with a wild gene. Furthermore, our analysis shows a statistically significant association between the LOF gene and higher ISR incidence (OR 95% CI = 2.71 [1.42–5.16], P = 0.003) ( Figure 2).

## Discussion

The response to clopidogrel differs from person to person. Many investigations have been conducted to determine the biological components involved in this variance. Polymorphisms in the liver cytochrome isozyme P450 (CYP2C19, CYP3A4, and CYP3A5), as well as the P2Y12 receptor itself, seem to be the major cause of variation. ^13^^,^^14^ The ability of the LOF gene to reduce the clopidogrel response in platelets was the first to be revealed, as seen by enhanced platelet aggregation in terms of CYP2C19 polymorphisms. ^13^

Numerous studies have found that CYP2C19 polymorphisms vary greatly among ethnic groups. In comparison to the white population (25%–35%) and the black population (35%–45%), the Asian population (55–70%) exhibited a greater prevalence rate of the LOF CYP2C19 allele variation (CYP2C19 *2 and *3). In comparison to the white population (18%), the Asian population (4%) exhibited a lower frequency of the CYP2C19 GOF variant allele (CYP2C19 *17). ^14^

A study by Mega *et al*. involving 1,477 acute coronary syndrome patients, with primary outcomes characterized by death from nonfatal myocardial infarct (MI), nonfatal stroke, and other cardiovascular causes, showed a significantly higher rate of LOF polymorphism (12.1% vs 8%; P = 0.01) than wild-type CYP2C19. ^15^ For 450 days after clopidogrel therapy, similar results were reported for the rate of stent thrombosis (2.6% vs 0.8%; P = 0.02). Another study of 259 patients using clopidogrel medication for one month after their first MI found that individuals with CYP2C19 polymorphisms had considerably poorer five-year survival (HR = 3.69; 95% CI = 1.69–8.5; P = 0.0005), which was linked to considerably poorer clinical outcomes following coronary stenting. ^16^ In another study, 552 individuals were revealed to have the wild-type CYP2C19 allele, and 245 had at least one of two alleles. Patients who were homozygous for the wild-type gene had considerably lower platelet aggregation levels than those with two alleles (11% vs 23%; P < 0.0001). ^17^

This meta-analysis is the first to provide a comprehensive review of the existing evidence from observational studies, which assesses the association between CYP2C19 LOF gene polymorphisms and the risk of ISR. The study offers a novel pooled estimate of this relationship, showing a significant association between ISR and the LOF gene. The odds of ISR were found to be 2.71 times higher in the LOF gene compared to the wild gene. The wild gene can metabolize clopidogrel more effectively than the LOF gene. Zhang *et al*. and Nozari *et al*. stated that they used clopidogrel 75 mg in their study. ^8^^,^^10^ Moreover, Zhang *et al*. also had a third group of patients with the LOF gene who were administered a double dose of clopidogrel (150 mg). There were no significant differences in this group compared to patients with the wild gene who were given a normal dose of clopidogrel (75 mg). ^8^

The first choice for a clopidogrel resistance management strategy is to increase the dose of clopidogrel, with the current loading dose for clopidogrel being 300 mg. A larger loading dosage of 600 mg was compared to a lower loading dosage of 300 mg in two studies. Cuisset *et al*. randomly selected 292 patients undergoing stenting for non-ST elevation myocardial infarction (STEMI) to receive a loading dose of either 300 mg or 600 mg 12 hours before percutaneous coronary intervention (PCI). One month after the intervention, all patients were administered aspirin 160 mg and clopidogrel 75 mg daily. The 600 mg loading dosage group demonstrated reduced adenosine diphosphate (ADP) induced platelet aggregation and P-selection expression compared to the 300 mg loading dosage group.

Furthermore, there were fewer cardiovascular incidents in the 600 mg loading dosage group after one month (7 vs 18 events; P = 0.02). ^18^ One hundred and forty-eight patients undergoing elective PCI were randomly assigned to three groups in a study conducted by L’Allier *et al*. Group A patients were administered 75 mg clopidogrel on the morning of the procedure and 300 mg clopidogrel a day (≥15 hours) before the procedure. Group B patients were administered 600 mg clopidogrel in the morning (≥2 hours) of the procedure. Meanwhile, group C patients were administered 600 mg clopidogrel on the morning of the procedure and 600 mg clopidogrel a day before the procedure. Group C revealed substantially higher relative inhibition of peak and final ADP-stimulated platelet aggregation than groups A and B. Thus, it appears that a successive 600 mg/dose of clopidogrel bolus resulted in more substantial platelet inhibition than a single loading dosage. ^19^

The effectiveness of a daily maintenance dosage of clopidogrel 150 mg in patients undergoing elective PCI was examined in a study by Angiolillo *et al*. Both groups of patients continued medication for 30 days before returning to the regular dose. ADP-induced platelet aggregation (20 μM) was higher in 75 mg clopidogrel/day patients than in 150 mg clopidogrel/day individuals (64% vs 52.1%; P < 0.001). ^20^ The VerifyNow test evaluated relative platelet aggregation in response to 5 μM ADP (45.1% vs 65.3%; P < 0.001), and platelet function inhibition (60 vs 117; P = 0.004) was substantially improved in patients who received clopidogrel 150 mg compared to those given 75 mg. ^20^

A larger trial of 153 individuals with a low clopidogrel response (platelet reactivity index 69%) to 75 mg/day or 150 mg/day (n = 95 or n = 58) clopidogrel found similar results. After two weeks of therapy, clopidogrel 150 mg/day was linked with a considerably lower platelet reactivity index than 75 mg/day (43.9% vs 58.6%; P < 0.001). After switching to clopidogrel 150 mg/day for two weeks, 20 out of 31 patients in the 75 mg/day group were responsive (platelet reactivity was 69%). ^21^ Overall, the outcomes of this study point to the possibility of employing higher clopidogrel bolus and maintenance dosages.

Other alternative drugs include prasugrel, ticagrelor and cilostazol. Although there is limited evidence to suggest that using prasugrel interacts with proton pump inhibitors (PPIs) via cytochrome P450, a study of TRITON-TIMI 38 and PRINCIPLES 44 showed that PPIs did not affect the efficacy of prasugrel. Thus, the use of PPIs with prasugrel is preferable. Ticagrelor is a new platelet aggregation inhibitor. Ticagrelor, like clopidogrel, binds to P2Y12, which acts as an antagonist to the ADP receptor on platelets, preventing platelet aggregation.

Unlike clopidogrel, ticagrelor reversibly binds to P2Y12 and does not displace ADP from the receptor, allowing it to target 2-MeS-ADP-induced signalling. Furthermore, because ticagrelor does not need hepatic enzymatic activation, it is more consistent in reducing platelet aggregation. It has a lower risk of medication interactions and is not affected by CYP polymorphisms. ^22^

Cilostazol suppresses cAMP degradation in platelets by inhibiting platelet aggregation via phosphodiesterase-3-blockers. As a result, cilostazol may be an option for people who are resistant to clopidogrel. In patients receiving contemporary stent-based percutaneous procedures, using cilostazol in combination with clopidogrel and aspirin may minimize restenosis cerebral and cardiac side effects. ^23^ As a result, cilostazol is more beneficial than clopidogrel at high maintenance dosages. However, cilostazol was terminated more often in this trial due to the adverse effects.

Shim *et al*. randomly categorized 400 patients who underwent successful PCI with DES into triple antiplatelet therapy (clopidogrel, cilostazol, and aspirin) and dual antiplatelet therapy (clopidogrel and aspirin) groups. They reported a significant decrease in clopidogrel resistance in the triple antiplatelet group (19.7% vs 40%, P < 0.001). As a result, the rate of clopidogrel resistance in patients undergoing PCI with DES can be reduced. ^24^ Several trials compared the effectiveness and safety of using cilostazol with aspirin and clopidogrel in dual antiplatelet therapy (DAPT). Cilostazol combination therapy has significant advantages in major cardiac side effects, death from any cause, cardiac death, target lesion revascularization, and in-segment restenosis. Benefits are reported in patients with clopidogrel resistance. ^23^

Several limitations were evident in the present study. First, regarding the association between the LOF gene and ISR, there were limited studies with small sample sizes, which may have limited statistical power. Second, none of the studies included were RCTs. Third, varying types of LOF genes were included in the studies, and some studies did not state clearly whether they were examining homozygote LOF genes or heterozygote LOF genes. Fourth, the studies utilized different stent types and did not clearly state the percentage of BMS and DES used in their studies, as we know that BMS have a higher tendency to form ISR. Fifth, none of the studies used intracoronary imaging. Furthermore, individual patient-level data were not available.

## Conclusions

In conclusion, the present study supports the theory that, in patients who have undergone coronary stenting and been treated with clopidogrel, the presence of the LOF gene increases their likelihood of ISR. Furthermore, larger-scale studies are required to examine appropriate strategies in patients with the LOF gene who have undergone coronary stenting.

## Data availability

### Underlying data

All data underlying the results are available as part of the article and no additional source data are required.

### Reporting guidelines

Figshare: PRISMA checklist and Funnel plot for ‘CYP2C19 polymorphism and coronary in-stent restenosis: A systematic review and meta-analysis’. https://doi.org/10.6084/m9.figshare.19312115. ^11^

Data are available under the terms of the Creative Commons Attribution 4.0 International license (CC-BY 4.0)
