# Supplementary material for: CYP2C19 polymorphism and coronary in-stent restenosis: A systematic review and meta-analysis
Source: F1000Res. 2025 Jan 15;11:346. Originally published 2022 Mar 22. [Version 3] doi: 10.12688/f1000research.109321.3 (PMC11078198; doi:10.12688/f1000research.109321.3)
Supplement: Peer review history

# Reviewer report: Reviewer response for version 2

**DOI:** 10.5256/f1000research.134740.r249958

**Published:** 2024-05-06

**Authors:** Fazli Rabbi Awan

**Affiliations:** 1 Diabetes and Cardio-Metabolic Disorders Laboratory, Health Biotechnology Division, National Institute for Biotechnology and Genetic Engineering, Faisalabad, Pakistan; 2 Pakistan Institute of Engineering and Applied Sciences, Islamabad, Pakistan

I have read this article with great interest. It is well written study. Though, the sample size (number of papers used in this meta-analysis) was small as less literature has been available, authors were careful to draw any generalized conclusions. They have hinted towards ISR correlatoin with the risk (loss of function) genotype of the investigated gene.

 Overall, it is a good read.

 I only found one typing mistake:

 P-selection should be P-selectin

 and either use, vs or verses throughout.

Are the rationale for, and objectives of, the Systematic Review clearly stated?

Yes

Is the statistical analysis and its interpretation appropriate?

I cannot comment. A qualified statistician is required.

If this is a Living Systematic Review, is the ‘living’ method appropriate and is the search schedule clearly defined and justified? (‘Living Systematic Review’ or a variation of this term should be included in the title.)

Not applicable

Are sufficient details of the methods and analysis provided to allow replication by others?

Yes

Are the conclusions drawn adequately supported by the results presented in the review?

Yes

Reviewer Expertise:

Genetics of Metabolic and Cardiovascular Disorders

I confirm that I have read this submission and believe that I have an appropriate level of expertise to confirm that it is of an acceptable scientific standard.

# Response

**Published:** 2025-01-03

**Authors:** Ivana Purnama Dewi

**Affiliations:** Airlangga University, Indonesia

Thank you for your thorough review and valuable feedback on our manuscript. We appreciate your positive comments and constructive suggestions.

 I only found one typing mistake:

 P-selection should be P-selectin

 and either use, vs or verses throughout.

**Authors response:** The typo "P-selection" has been corrected to "P-selectin." We sincerely apologize for this oversight.

 Additionally, we have carefully reviewed the manuscript and ensured that "vs" is used consistently throughout the text.

# Reviewer report: Reviewer response for version 2

**DOI:** 10.5256/f1000research.134740.r249956

**Published:** 2024-03-23

**Authors:** Vaiva Lesauskaitė, Renata Paukstaitiene

**Affiliations:** 1 Laboratory of Molecular Cardiology, Institute of Cardiology, Lithuanian University of Health Sciences, Kaunas, Lithuania; 2 Department of Physics, Mathematics and Biophysics, Lithuanian University of Health Sciences, Kaunas, Lithuania

1. There is no information from which year publications were searched for systematics literature review in the search strategy.

 2. Authors chose odds ratio as the effect size. But they use the terms “correlation” and “risk” in  the results sections. No statistical methods were used to calculate correlation or risk. Terms must be corrected according to used statistical methods. 

 3. Two models (Fixed effect model and random effect model) are mentioned in the Methods chapter, but only the results of fixed effect are presented in the Results chapter. Why random effect model was not included  in the results?.

 4. The use of the fixed-effect model for the analysis is not acceptable because the analyzed results are gained from different types of studies, and studies are taken from different countries. Authors indicated that choice of fixed- effect model was based on the absence of heterogeneity. But only four studies are included in the meta- analysis, thus the power of the test of heterogeneity is low. Thus, authors don’t give sufficient evidence that the studies really represent a single population, and all studies measure the same effect, which is different due to sampling error.

 5. To edit Fig.5. It is not clear which group is assigned as a treatment group in the research. Usually, the same side for treatment and control group is used in the table and in the Forest plot. Authors changed these sides, and it is not clear if the conclusion is correct.

 6. Newcastle – Ottawa Scale is used to assess the quality of the publication in the systematic literature review, but it is not suitable for estimation of the publication bias for the meta-analysis as it is indicated in the abstract of the review. At least Funnel plot has to be presented to show that there is no publication bias.

 6. Table 2 line Age  - it is not clear what numerical characteristics are presented “?±?” mean±SD?, SE?

 7. The statement "The odds of ISR were 2.8% higher in the LOF gene compared to the wild gene" is not correct. OR 2,8 means increase in 2,8 folds or 280 percents.

 8.The novelty and significance of meta analysis results are not disclosed in Discussion

Are the rationale for, and objectives of, the Systematic Review clearly stated?

Yes

Is the statistical analysis and its interpretation appropriate?

No

Are sufficient details of the methods and analysis provided to allow replication by others?

No

Are the conclusions drawn adequately supported by the results presented in the review?

Yes

Reviewer Expertise:

Cardiovascular pathology, pharmacogenomics;,

We confirm that we have read this submission and believe that we have an appropriate level of expertise to confirm that it is of an acceptable scientific standard, however we have significant reservations, as outlined above.

# Reviewer report: Reviewer response for version 1

**DOI:** 10.5256/f1000research.120809.r128371

**Published:** 2022-04-04

**Authors:** Dyana Sarvasti

**Affiliations:** 1 Department of Internal Medicine, Faculty of Medicine, Widya Mandala Catholic University, Surabaya, Indonesia

This meta-analysis study is interesting and useful, although it only involves four non-randomized controlled trials. It can be used as additional knowledge about the management of antiplatelet therapy use in patients undergoing PCI who are suspected of having the presence of the CYP2C19 LOF gene. Unfortunately, the research objectives related to the use of antiplatelets are not fully described in the abstract, even though in the discussion and conclusions there are many mentions of the relationship between LOF genes, ISR, and various types of antiplatelets, especially clopidogrel. If possible, the association of the CYP2C19 LOF gene with antiplatelet use (clopidogrel) should also be addressed in the research objectives in the abstract.

 In addition, there are some mistypes that need to be corrected. Here are some points related to this:

1. In table 2, it appears that “ **Cross sectional”** is missing a hyphen. Consider adding the hyphen(s).
2. In abbreviations in table 2, It appears that “ **bare metal**” is missing a hyphen. Consider adding the hyphen(s).
3. In abbreviations in table 2, It appears that “ **drug eluting**” is missing a hyphen. Consider adding the hyphen(s).
4. There is an inconsistency in writing the title of the bibliography in the reference list. Sometimes it is written in lower case, sometimes in upper case. Consider making corrections.

Thank you for your kind attention.

Are the rationale for, and objectives of, the Systematic Review clearly stated?

Partly

Is the statistical analysis and its interpretation appropriate?

Yes

Are sufficient details of the methods and analysis provided to allow replication by others?

Yes

Are the conclusions drawn adequately supported by the results presented in the review?

Yes

Reviewer Expertise:

Cardiovascular Prevention and Rehabilitation

I confirm that I have read this submission and believe that I have an appropriate level of expertise to confirm that it is of an acceptable scientific standard.

# Response

**Published:** 2022-06-02

**Authors:** Ivana Purnama Dewi

**Affiliations:** Airlangga University, Indonesia

Dear Dyana Sarvasti, M.D

 Thank you for your kind review

 We will make some corrections regarding your suggestions

 Best regards,

 Yusra Pintaningrum, M.D
